# Patient perspectives on telemedicine during the COVID-19 pandemic: a mixed-methods community-based study

**DOI:** 10.1186/s12913-023-09794-w

**Published:** 2023-07-27

**Authors:** Marije J. Splinter, M. Kamran Ikram, Charles W. Helsper, Patrick J.E. Bindels, Evelien I.T. de Schepper, Silvan Licher

**Affiliations:** 1 Department of Epidemiology, Erasmus MC - University Medical Center Rotterdam, PO Box 2040, Rotterdam, 3000 CA The Netherlands; 2 Department of Neurology, Erasmus MC - University Medical Center Rotterdam, Rotterdam, The Netherlands; 3 Julius Center for Health Sciences and Primary Care, University Medical Center Utrecht, Utrecht University, Utrecht, The Netherlands; 4 Department of General Practice, Erasmus MC - University Medical Center Rotterdam, Rotterdam, The Netherlands

**Keywords:** Telemedicine, Cohort study, Mixed-methods, Health services research

## Abstract

**Background:**

Detailed community-based perspectives on patient experiences with telemedicine are currently lacking, yet essential to assess clinical applicability of telemedicine during and beyond pandemics, alike COVID-19. The aim of this study was to expose patient perspectives on virtual compared to in-person consultations, including determinants of these preferences.

**Methods:**

We invited 5864 participants of the population-based Rotterdam Study to fill in a validated questionnaire using both close-ended and free-text questions. The questionnaire was sent on 30 July 2020, following a period of lockdowns and closures of non-essential workplaces. It assessed preferences for physician contact, healthcare utilisation, socioeconomic factors, and overall health. Those who experienced at least one virtual consultation (telephone or video call) between March 2020 and the beginning of July 2020 were asked whether those consultations were more, equally or less pleasant than in-person consultations, and to detail their experiences through free-text comments. These narrative data were examined using thematic analysis.

**Results:**

4514 participants completed the questionnaire (response rate 77.0%, 58.7% women, mean age 70.8 ± 10.5 years). 1103 participants (24.4%) reported having had experience with virtual consultations. Half of these participants considered virtual consultations less pleasant than in-person consultations (N = 556; 50.4%), while 11.5% found it more pleasant. In total, we coded free-text comments of 752 participants. Prominent themes behind patient preferences for virtual or in-person consultations were lack of nonverbal communication, lack of physical examination, consultation scheduling, personal circumstances, and the presence of somatic and/or language barriers.

**Conclusions:**

Based on the experiences of a large elderly patient population, we showed that preference for virtual or in-person consultations is dependent on personal and situational variety, and their interplay. Healthcare providers should consider patients’ complex care needs and evaluate the potential added value of nonverbal communication and physical examination before scheduling a virtual consultation.

**Supplementary Information:**

The online version contains supplementary material available at 10.1186/s12913-023-09794-w.

## Introduction

The COVID-19 pandemic in the beginning of 2020 led to a rapid shift from in-person to virtual care throughout the entire healthcare system, as it limited the risk of becoming infected with COVID-19 while enabling continued access to medical care [1–3]. Several studies have since then assessed the attitudes of healthcare providers towards telemedicine and their willingness to incorporate these systems in clinical practice, during but also beyond the pandemic [4–7]. From a physician’s viewpoint, advantages of virtual healthcare are the ability to reach out to patients who are unable to visit a healthcare institution and to monitor patients’ health in a home-based setting, therefore, enhancing patient-centred care [4–6]. However, physicians also expressed their concerns about the risk of misdiagnosis because of the absence of certain routine medical procedures, including physical examination, which would hinder clinical decision making [4, 7].

In contrast, population-level data on patient perspectives on telemedicine are scarce, as most scientific evidence is focused on effectiveness of telemedicine in specific care settings instead of unravelling patients’ personal experiences [8, 9]. For instance, telemedicine may be able to reduce short-term cardiovascular-related hospitalisation and mortality risk among patients with heart failure [10]. Studies that did focus on the patient’s viewpoint are limited as the majority based their findings on general measures of satisfaction [11–14]. Existing evidence should be complemented with the narratives and detailed opinions of a general patient population, as such knowledge is key to successful healthcare wide implementation of this approach to care provision.

Therefore, in this community-based study among community-dwelling patients, we aimed to expose perspectives on virtual compared to in-person consultations, including the determinants of these preferences, using a mixed-methods approach.

## Methods

### Study population: the Rotterdam Study

This study was conducted within the community-based Rotterdam Study, an ongoing prospective cohort study designed to investigate the aetiology, preclinical course, natural history, and potential targets for intervention for chronic diseases in mid- and late-life [15]. The Rotterdam Study was initiated in 1990 (RS-I) among residents of the district Ommoord in Rotterdam, the Netherlands, and has been expanded in 2000 (RS-II), 2006 (RS-III) and 2016 (RS-IV). Currently, it comprises a total of 17,931 participants who were ≥ 40 years at study entry and were followed-up every 3 to 6 years. This study is reported according to both the Standards for Reporting Qualitative Research (SRQR) and Strengthening the Reporting of Observational Studies in Epidemiology (STROBE) guidelines.

### COVID-19 substudy

From April to October 2020, we sent six COVID-19 questionnaires to participants of the Rotterdam Study, with the aim of addressing various healthcare consequences of the pandemic, including the following aspects: COVID-19-related symptoms and risk factors; socioeconomic factors; medication use; lifestyle and mental health; and healthcare utilisation. All noninstitutionalised participants received the first and second questionnaire (N = 8732), whereas follow-up questionnaires were only sent to those who specifically indicated that they wanted to continue participating in this substudy. A detailed description of the methods including validation of the questionnaires has been reported elsewhere [16].

### Primary outcome: experiences with virtual consultations

In the fifth COVID-19 questionnaire, we particularly inquired participants about their experiences with virtual in comparison to in-person consultations. This questionnaire was sent to 5864 participants on 30 July 2020, following a period of strict lockdowns and other countermeasures to contain the spread of the virus [16]. We asked participants to report to what extent pre-existing or new healthcare consultations had been changed from in-person to virtual (telephone or video) consultations from the onset of the pandemic in March until the beginning of July 2020 (none; a minority; all consultations; not applicable). Subsequently, all participants who reported that they have had experience with virtual consultations were posed the following statement: “I considered virtual consultations more/equally/less pleasant than in-person consultations, because…”. The open-ended format of the question gave participants the opportunity to detail their experiences by filling in free-text comments.

### Potential determinants of consultation preferences

Besides age and sex (man; woman), we included the following determinants in the fifth questionnaire: incident chronic diseases (newly developed after filling in the previous COVID-19 questionnaire), the physician responsible for regular check-ups of chronic diseases (general practitioner; medical specialist; both; other), concern about contracting COVID-19 (never; rarely; sometimes; often; almost continuously, in the 14 days prior to filling in the questionnaire), and quality of life (scale 1–10: 1 = abominable, 10 = excellent, in the 14 days prior to filling in the questionnaire). From the first COVID-19 questionnaire, collected between April and June 2020, we derived occupational status (working; on sick leave; unemployed; retired; other) and baseline prevalence of chronic conditions (such as cancer; heart disease; stroke; chronic lung disease; neurodegenerative disease; diabetes; mental illness) [16]. Participants’ educational level (primary education; low/intermediate general of lower vocational; intermediate vocational or higher general; higher vocational or university) was retrieved from pre-pandemic measurements in 2015 (RS-I, II and III) and 2020 (RS-IV), and categorised according to the International Standard Classification of Education (ISCED) by the United Nations Educational, Scientific, and Cultural Organization (UNESCO) [17].

### Data analysis

Descriptive analyses included categorical variables, presented as proportions (numbers, %), and continuous data, presented as means and standard deviations. Subgroup analyses comprised mean differences for varying demographic characteristics for consultation type preferences (1 = preference for in-person consultations; 2 = equally pleasant; 3 = preference for virtual consultations) using Pearson’s ${X}^{2}$ test for categorical variables and one-way ANOVA for continuous variables. Subsequently, we employed age- and sex-adjusted ordinal logistic regression analyses to assess determinants of preference for consultation type. A p-value of less than 0.05 was considered statistically significant.

The narrative, free-text data were analysed inductively through thematic analysis in accordance with the guidelines by Braun and Clarke using ATLAS.ti version 22.0 [18]. First, one researcher retyped the comments from SPSS to ATLAS.ti and read them several times in order to become familiar with the content and get a sense of potential patterns. Then, this researcher coded the entire document independent from another member of the research team who coded a random subsample of fifty comments. These two sets of codes were compared, refined and merged if applicable, after which they were sorted into candidate themes, which were discussed in consensus meetings with two other members of the research team. This resulted in the redefining and naming of the final sub- and main themes. We focused on generating themes that cohered together meaningfully, but also had clear and identifiable distinctions [18].

As a sensitivity analysis, we stratified perspectives on virtual in comparison to in-person consultations between participants who reported a history of cardiovascular diseases (any; heart attack; narrowing of arteries in the legs; stroke/TIA; other) or cancer to examine whether their preferences differed from individuals who were free of any of these conditions at the time of filling in the questionnaire. Quantitative data were handled and analysed with the Statistical Package for the Social Sciences software (SPSS), version 25.0, and R version 4.0.5.

### Researcher characteristics and reflexivity

The first and senior author of this study (MJS and SL) independently coded the free-text data. Both have a background in epidemiology, either with a major in public health epidemiology (MJS) or clinical epidemiology (SL). Additionally, MJS holds a MSc in sociology. SL obtained a PhD as well as an MD and is a general practitioner in training. The other co-authors, CWH, PJEB, and EITS, have a background in general practice, whereas MKI has a background in neurology and epidemiology. Data collection for this study took place in August 2020, coinciding with the onset of the second wave of COVID-19 in the Netherlands (July – October 2020). During this period, COVID-19 infections were gradually increasing. Countermeasures to contain the spread of the virus were limited to social distancing, mask-wearing, and other basic hygienic measures. While consultation rates in primary, secondary, and tertiary care were similar to previous years, there was an ongoing backlog in elective and non-urgent medical care [19].

## Results

### Characteristics

4514 out of 5864 participants returned the questionnaire (response rate 77.0%), of which the majority was received within two weeks of being sent out (N = 3842, 85.1%). Non-responders to the questionnaire (N = 1350) were slightly more often women (60.7% vs. 57.4%), lower educated (7.6% vs. 5.6% primary education) and older (mean age 73.8 ± 9.2 vs. 70.3 ± 11.1 years) in comparison with responders (Supplementary Table 1). The final study population consisted of 1103 (24.4% of respondents) participants who indicated that at least one of their regular or new consultations had been conducted virtually between March and the beginning of July 2020. Most of these participants were women (58.7%), relatively higher educated (29.7% higher vocational or university level vs. 4.9% primary education) and likely to have a history of any chronic disease (82.4%) (Table 1). Among those with a chronic disease, the majority indicated either being followed-up by a medical specialist, or by both the general practitioner and medical specialist. Those who did not have experience with virtual consultations (N = 3411) were less likely to be retired (60.5% vs. 65.0%), less concerned about contracting COVID-19 (16.2% vs. 6.7% never concerned), and less often had a chronic disease (56.7% vs. 82.4%) (Supplementary Table 2).

**Table 1:** Characteristics of the study population, stratified by type of experience with telemedicine compared to in-person consultations (N = 1103). Values are numbers (percentages) unless stated otherwise

	Full sample (N = 1103)	More pleasant	Equally pleasant	Less pleasant	p-value
**Sex**					0.217
Men	456	48 (10.5)	164 (36.0)	244 (53.5)	
Women	647	79 (12.2)	256 (39.6)	312 (48.2)	
**Age (years)**					0.545
Mean, SD	70.8 (10.5)	71.5 (11.6)	70.4 (10.3)	70.9 (10.3)	
**Age (categories)**					0.309
< 65 years	302	32 (10.6)	124 (41.1)	146 (48.3)	
65–79 years	551	58 (10.5)	205 (37.2)	288 (52.3)	
≥ 80 years	250	37 (14.8)	91 (36.4)	122 (48.8)	
**Type of questionnaire**					< 0.001
Paper	638	84 (13.2)	218 (34.2)	336 (52.7)	
Digital	465	43 (9.2)	202 (43.4)	220 (47.3)	
**Educational level**					0.360
Primary education	54	5 (9.3)	25 (46.3)	24 (44.4)	
Low/intermediate general or lower vocational	352	48 (13.6)	129 (36.6)	175 (49.7)	
Intermediate vocational or higher general	357	42 (11.8)	144 (40.3)	171 (47.9)	
Higher vocational or university	328	32 (9.8)	117 (35.7)	179 (54.6)	
Missing	12	0	5	7	
**History of any chronic disease**					0.987
No	153	18 (11.8)	59 (38.6)	76 (49.7)	
Yes	909	106 (11.7)	345 (38.0)	458 (50.4)	
Missing	41	3	16	22	
**Physician who provides regular check-ups for chronic disease**					0.401
General practitioner	205	23 (11.2)	79 (38.5)	103 (50.2)	
Medical specialist	339	29 (8.6)	129 (38.1)	181 (53.4)	
General practitioner and medical specialist	219	26 (11.9)	80 (36.5)	113 (51.6)	
Other	27	6 (22.2)	10 (37.0)	11 (40.7)	
Missing	15	1	4	14	
**Multimorbidity**					0.386
Two chronic diseases	306	44 (14.4)	102 (33.3)	160 (52.3)	
Three or more chronic diseases	297	30 (10.1)	116 (39.1)	151 (50.8)	
Missing	38	3	14	21	
**Current occupational status**					0.478
Working	197	23 (11.7)	83 (42.1)	91 (46.2)	
On sick leave	22	2 (9.1)	9 (40.9)	11 (50.0)	
Unemployed	33	7 (21.2)	10 (30.3)	16 (48.5)	
Retired	717	85 (11.9)	267 (37.2)	365 (50.9)	
Other	77	5 (6.5)	28 (36.4)	44 (57.1)	
Missing	57	5	23	29	
**Concerns about contracting COVID-19**					0.091
Never	74	5 (6.8)	33 (44.6)	36 (48.6)	
Rarely	299	35 (11.7)	127 (42.5)	137 (45.8)	
Sometimes	582	63 (10.8)	216 (37.1)	303 (52.1)	
Often	115	20 (17.4)	31 (27.0)	64 (55.7)	
Almost continuously	25	3 (12.0)	9 (36.0)	13 (52.0)	
Missing	8	1	4	3	
**Self-appreciated quality of life (1 = abominable, 10 = excellent)**					< 0.001
Mean, SD	7.1 (1.4)	7.3 (1.2)	7.3 (1.3)	6.9 (1.6)	
Missing	14	2	3	9	

N = number of participants; SD = standard deviation; COVID-19 = Coronavirus Disease 2019

### Virtual versus in-person consultations

**Fig. 1:**
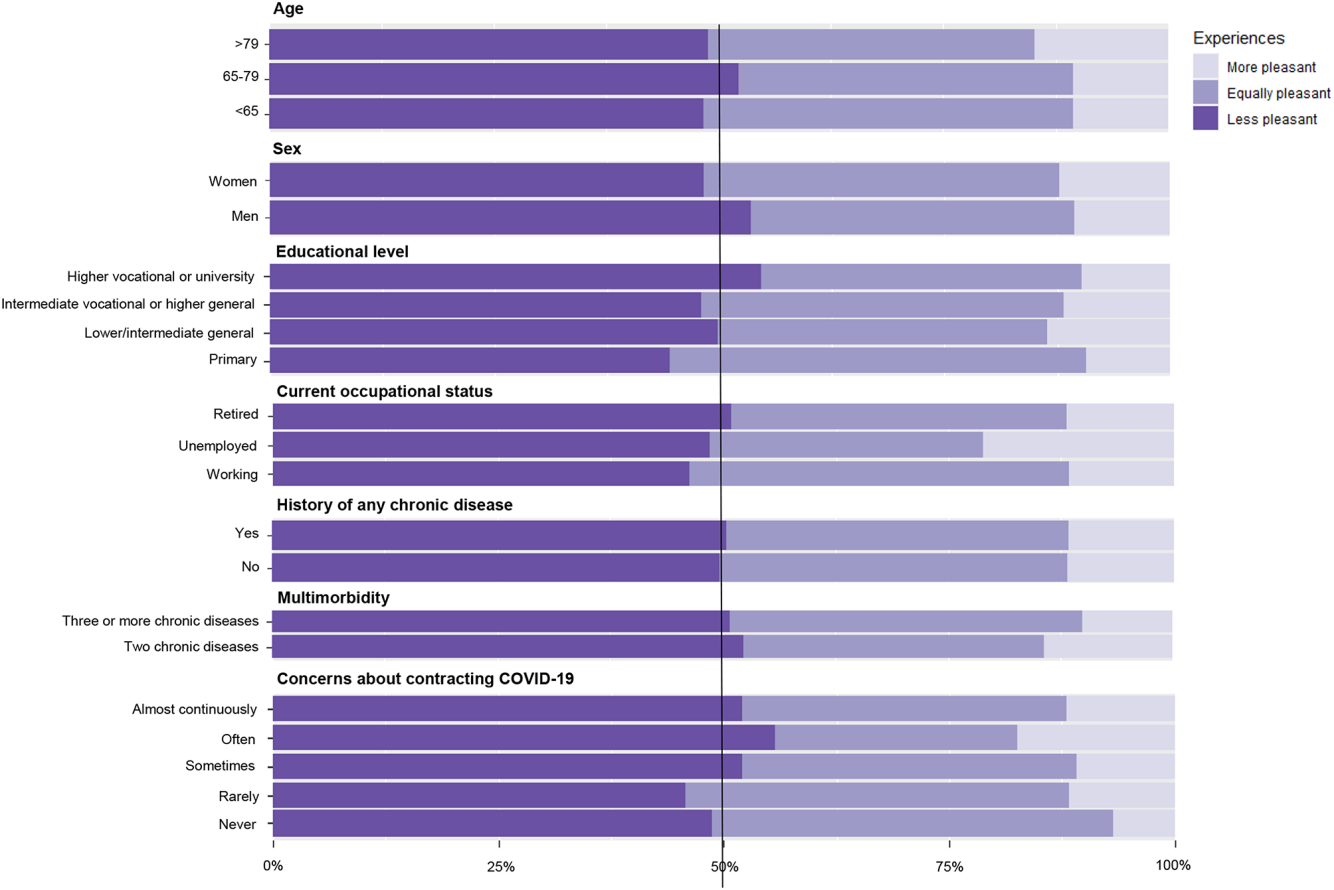
Experiences with virtual consultations compared to in-person consultations. COVID-19 = Coronavirus Disease 2019. Bold vertical line indicates 50% of participants within subgroups

About half of all participants (N = 556, 50.4%) considered virtual consultations less pleasant than in-person consultations, in contrast to 11.5% who viewed it as more pleasant (N = 127). 420 participants did not have a particular preference for either type of consultation (38.1%) (Table 1). Individuals who favoured virtual above in-person consultations were more likely to be of older age (14.8% of all older than 80 years vs. 11.2% of all younger than 65 years) (Table 1; Fig. 1). Experiences of participants with a history of cardiovascular diseases or cancer did not differ from participants free of those diseases (Supplementary Table 3).

In age- and sex-adjusted analyses, increased quality of life was the only determinant that was significantly associated with higher ORs of favouring virtual above in-person consultations (per level increase on a scale of 1 to 10: OR 1.23 [95% CI 1.13 to 1.34]) (Table 2). Negative effect estimates were shown for higher educational level (0.93 per level increase [0.81 to 1.06]) and increased concerns about contracting COVID-19 (0.92 per level increase [0.80 to 1.06]), however, these associations were not statistically significant.

**Table 2:** Determinants of patient experiences with telemedicine (N = 1103)

		OR (95% CI)
Age, per 10 years increase^a^		1.01 (0.90 to 1.13)
Women^b^		1.23 (0.97 to 1.55)
Educational level, per level increase		0.93 (0.81 to 1.06)
Current occupational status vs. employed	Retired	0.74 (0.48 to 1.14)
	Unemployed	1.11 (0.55 to 2.25)
History of any chronic disease, yes		1.03 (0.74 to 1.43)
Multimorbidity vs. no history of chronic diseases	Two chronic diseases	1.02 (0.72 to 1.45)
	Three or more chronic diseases	1.09 (0.76 to 1.56)
Concerns about contracting COVID-19, per level increase		0.92 (0.80 to 1.06)
Self-appreciated quality of life, per level increase		1.23 (1.13 to 1.34)*

CI = confidence interval; N = number of participants; OR = odds ratio;

COVID-19 = Coronavirus Disease 2019.

^a^ adjusted for sex.

^b^ adjusted for age.

* *p* < 0.001.

### Thematic analysis

814 out of 1103 (73.8%) participants left a free-text comment in which they reflected on their experiences with virtual consultations. We excluded 62 of these comments because they were either unclear or unrelated to the question. This resulted in 752 comments being available for analysis. Demographic characteristics of participants who solely responded to the question about experiences with virtual versus in-person consultations were comparable to those who also left a free-text comment, except for the fact that the latter group was slightly higher educated (33.4% vs. 29.7% higher vocational or university level) and more often had an occupation (20.7% vs. 17.9% working) (Supplementary Table 4). All (sub)themes and corresponding quotes are presented in Supplementary Table 5. In the following section we briefly summarise patients’ perspectives within each (sub)theme.

### Less pleasant

#### Theme 1: lack of nonverbal communication (N = 233)

Participants considered the absence of personal contact a major disadvantage of virtual consultations. They particularly mentioned requiring the ability to make eye contact and interpret body posture, speech patterns and facial expression during a consultation. Participants viewed nonverbal communication as an important source of information aiding them in understanding the content of the consultation and generating a natural conversation, whereas virtual consultations were experienced as more rigid.

#### Theme 2: lack of physical examination (N = 127)

For several participants, physical examination was part of their regular check-ups, where their blood pressure is measured, or their physical symptoms are inspected. However, during a virtual consultation, they had to depend on their own verbal descriptions of their signs and symptoms. This made some participants wonder whether they failed to convey something that a healthcare provider would have noticed (better) or asked about during an in-person visit. Not being able to validate their symptoms through an objective measurement or rely on the physician’s visual assessment generated distrust among participants in the proposed diagnosis or treatment plan.

#### Theme 3: somatic and/or language barriers (N = 68)

Participants with hearing or speech difficulties, a language barrier, or concentration issues emphasised their discomfort with virtual consultations, since they particularly struggle with processing and expressing verbal information. As these perceived impairments generated feelings of embarrassment and lowered their assertiveness, they did not feel at ease to pose questions and have an effective conversation with the healthcare provider.

#### Theme 4: consultation scheduling (N = 53)

Participants commented that they often did not have any say in the specific time that the virtual consultation would take place. Instead, they were told that the healthcare provider could contact them at any moment of the day. For example, during a telephone consultation, they would either wait beside the phone, unable to carry on with their daily routine, or they would receive the call in an environment that was inconvenient for conducting a personal conversation. In the latter case, the consultation would take less time since participants were taken by surprise and not well prepared. Participants expressed their dissatisfaction with the fact that they had to conform to their physician’s schedule, rather than coming to an agreement about a time that is convenient for both.

#### Theme 5: acquaintance with physician (N = 15)

Some participants would prefer not to conduct a virtual consultation with a healthcare provider they did not meet in person before. They would rather not share confidential details about their personal life and medical history with a physician who felt like a complete stranger. Participants emphasised the need for an in-person introduction in order to create a trusting patient-provider relationship.

### Equally pleasant

#### Theme 1: similar quality (N = 55)

A number of participants did not notice clear differences between in-person and virtual consultations, since these consultations were experienced as equally clear and effective.

#### Theme 2: symptom-dependent (N = 40)

Several participants mentioned that a virtual consultation was equally sufficient as an in-person consultation, because they did not have any new or severe symptoms to discuss with their healthcare provider. If that would have been the case, they would have preferred an in-person consultation.

#### Theme 3: alternation preference (N = 19)

Some participants expressed wanting to receive the possibility to alternate between in-person and virtual consultations. For instance, they would be satisfied with a first consultation taking place in person in order to get to know the healthcare provider and come up with a treatment plan, after which the regular check-ups would be handled through the phone or a video call. Other patients preferred the other way around, starting with a virtual consultation after which it would be decided whether an in-person consultation would be required as well.

### More pleasant

#### Theme 1: consultation content (N = 140)

Occasionally, a consultation merely consisted of answering questions or discussing test results, without the necessity of a physical examination. In those cases, some participants preferred having the possibility to decide not having to meet the healthcare provider in person, but conducting the conversation virtually.

#### Theme 2: personal circumstances (N = 107)

For virtual consultations, it was appreciated that there was no need to leave the house and travel to a healthcare institution, often having to take a day off from work. This was specifically pleasant for patients with mobility issues and for those who experienced feelings of stress and anxiety when being in a healthcare environment. Additional practical considerations that were mentioned were the limited amount of waiting time and not having to pay for parking or public transport.

#### Theme 3: satisfaction with physician (N = 36)

Participants favoured virtual consultations during which their physician listened thoroughly, took time for questions that arose spontaneously, and showed that he or she was well-prepared and aware of the participant’s medical history. Others appreciated being able to get in contact with their doctor easily, without having to make a physical appointment and wait a few days or weeks before their questions could be addressed.

#### Theme 4: COVID-19 (N = 26)

During the COVID-19 pandemic, virtual consultations served as a replacement for non-urgent, elective physical care. This was viewed as a convenient, but temporary solution. Participants valued receiving medical attention and having a physician assessing their symptoms without the risk of becoming infected with the virus. Additionally, during virtual consultations, they were allowed to have a relative or friend sitting next to them in contrast to the strict regulations in most healthcare institutions prohibiting more persons in the consultation room than the physician and patient.

## Discussion

### Summary

We found that one out of every four individuals had experience with telemedicine during the first months of the COVID-19 pandemic. The vast majority of those who had a virtual consultation expressed a general preference for in-person consultations or considered in-person consultations at least as pleasant as virtual consultations. These preferences were mainly dependent on the potential added value of nonverbal communication and physical examination, the subject of the consultation, and the presence of somatic and/or language barriers hindering adequate virtual communication.

### Strengths and limitations

In this study, we managed to obtain in-depth insights into experiences with telemedicine from the viewpoint of community-dwelling individuals, complementing evidence from previous studies that was either discipline-specific or predominantly based on general measures of satisfaction. Several limitations should also be taken into account. First, this study was subject to the risk of observer bias, as with all studies containing thematic analysis. We have sought to limit this risk by coding the free-text comments with two independent coders. Second, our study population primarily consisted of relatively elderly individuals, which means that our findings may have limited generalisability to younger patient populations, These individuals are typically more accustomed to integrating digital technologies into their daily lives, and therefore, their experiences with telemedicine may differ from those of our study population. Moreover, the homogeneity in ethnic background among our participants raises similar considerations, suggesting that our findings may not fully capture the experiences of individuals with a migration background.Third, the proportion of individuals who favoured virtual above in-person consultations might have been an overestimation, given the fact that individuals who prefer an in-person consultation may have been more likely to not have any experience with virtual consultations and, therefore, be excluded from the analyses. Finally, we were not able to stratify perspectives based on the specific type of virtual consultation that participants encountered given the fact that we posed one question to inquire about both telephone and video consultations.

### Comparison with existing literature

Previous studies showed that the inability to conduct a physical examination is the primary concern of healthcare providers when implementing telemedicine in clinical practice [4, 7]. Our findings revealed that patients also considered these procedures a vital part of a medical consultation, worrying that the lack of physical examination will lead to significant symptoms being overlooked. This fear often resulted in distrust in the proposed medical treatment, which is associated with deteriorating health outcomes and poor medication adherence [20]. A potential solution to this issue could be remote monitoring of patients through external, wearable, or implantable devices such as blood pressure cuffs, thermometers, and glucometers [21]. However, we showed that not all patients are expected to benefit from this type of healthcare delivery, such as those with hearing, speech, or concentration difficulties, or those who experience language barriers. Consequently, a group of patients for whom telemedicine will not be able to replace an in-person consultation will remain, even if medical procedures would be largely similar.

Our findings did not reflect preferences for a particular type of consultation among the elderly and individuals with low socioeconomic status. In contrast, previous studies suggested discrepancies in uptake of digital health services among these groups of individuals, which were enhanced by the COVID-19 pandemic [22, 23]. Possibly, participants in our study mainly had experience with telephone consultations, which were embedded in clinical practice before the pandemic on a larger scale than video consultations were. Another potential explanation appeared from the thematic analysis, which showed that particularly visit-related instead of sociodemographic factors determined experiences with telemedicine, such as the clarity of patient-physician communication and the type of symptoms that participants experienced.

### Implications for research and/or practice

During the COVID-19 pandemic, the use of telemedicine expanded as it ensured safe access to medical care without requiring presence of both patient and physician in the same room. Even though the adoption of this type of healthcare delivery is expected to increase given its additional advantages beyond the pandemic, implementation should be done with caution and in consideration of the patients’ personal needs. Our findings emphasised that even groups of patients that are similar in sociodemographic characteristics can have diverse and complex care needs, affecting their ability and willingness to receive virtual or in-person care [24].

To enhance our understanding of the intricate dynamics associated with virtual healthcare delivery, we propose several suggestions for future research on telemedicine. First, stratifying on the specific platform as well as the type of healthcare provider who conducted the virtual consultation could provide a more detailed comprehension of patients’ experiences with telemedicine. Furthermore, a comparative analysis of experiences between urban and rural areas could be considered, given the fact that our study population consisted of residents from a single district. Lastly, investigating experiences with limited physical examination during video consultations could offer further insights into whether the challenges associated with virtual consultations in terms of physical examination can be overcome or mitigated in any manner.

## Conclusions

The preference for virtual or in-person consultations is dependent on both personal and situational variety, and their interplay. We showed that the majority of community-dwelling individuals considered virtual consultations an acceptable alternative, but not always an appropriate substitute for in-person medical care. Scheduling a virtual consultation in clinical practice should, therefore, be done in consideration of patients’ complex care needs, the potential added value of non-verbal communication and physical examination, and contextual personal preferences.

## Electronic supplementary material

Below is the link to the electronic supplementary material.

Supplementary Material 1

## Data Availability

The datasets generated and/or analysed during the current study are not publicly available due to legal and ethical restrains but are available from the corresponding author on reasonable request.
