# Correction: Patient perspectives on telemedicine during the COVID-19 pandemic: a mixed-methods community-based study

**DOI:** 10.1186/s12913-025-12302-x

**Published:** 2025-01-28

**Authors:** Marije J. Splinter, M. Kamran Ikram, Charles W. Helsper, Patrick J. E. Bindels, Evelien I. T. de Schepper, Silvan Licher

**Affiliations:** 1 Department of Epidemiology, Erasmus MC - University Medical Center Rotterdam, PO Box 2040, Rotterdam, 3000 CA The Netherlands; 2 Department of Neurology, Erasmus MC - University Medical Center Rotterdam, Rotterdam, The Netherlands; 3 Julius Center for Health Sciences and Primary Care, University Medical Center Utrecht, Utrecht University, Utrecht, The Netherlands; 4 Department of General Practice, Erasmus MC - University Medical Center Rotterdam, Rotterdam, The Netherlands

**Correction: BMC Health Serv Res 23, 803 (2023)**

**https://doi.org/10.1186/s12913-023-09794-w**

In this article an incomplete figure appeared as Fig. 1 due to a typesetting mistake. The headings above each group of graphs were missing.

Figure 1 (incorrect):

**Fig. 1:**
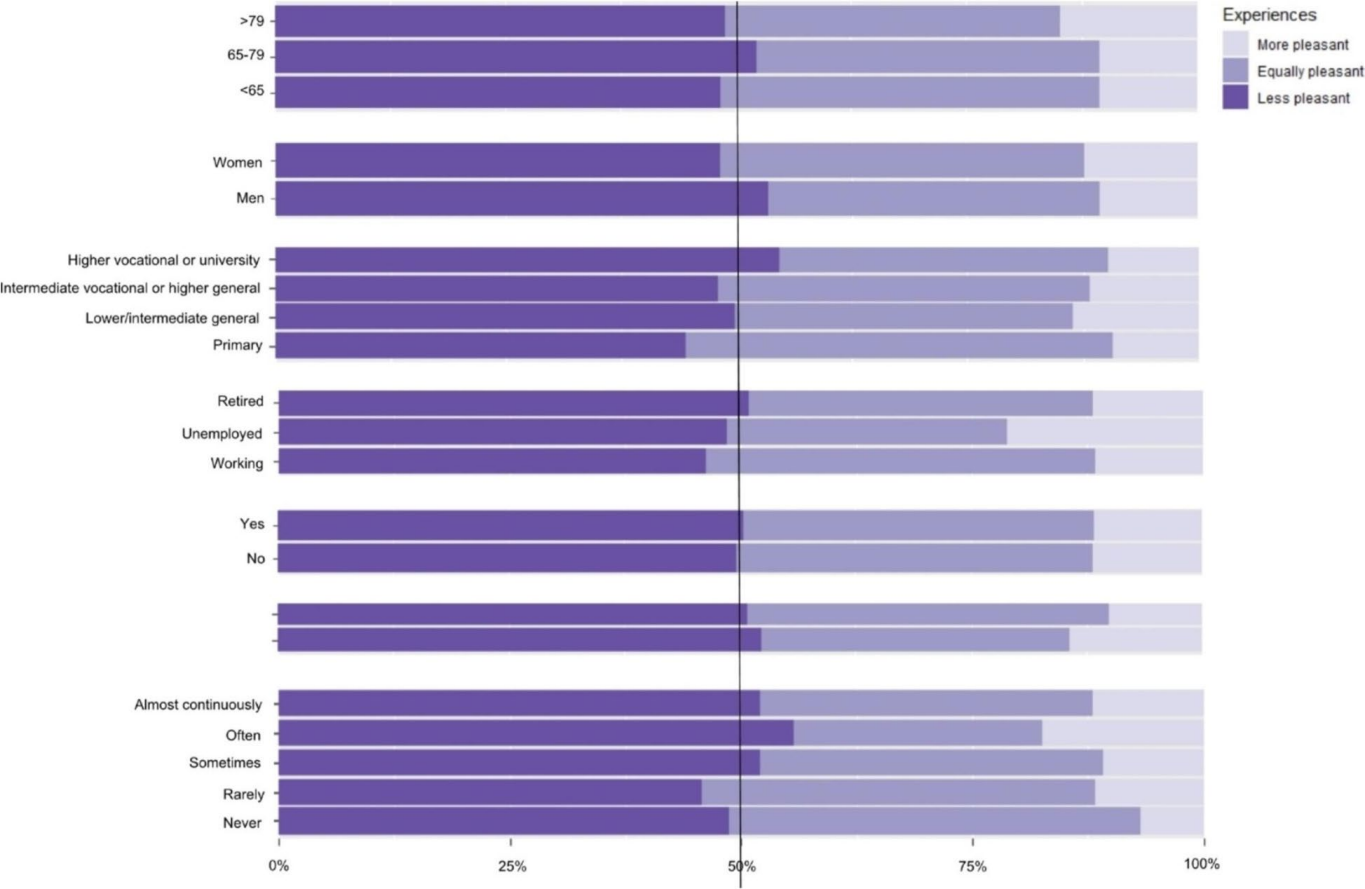
Experiences with virtual consultations compared to in-person consultations. COVID-19 = Coronavirus Disease 2019. Bold vertical line indicates 50% of participants within subgroups

Figure 1 (correct):

**Fig. 1:**
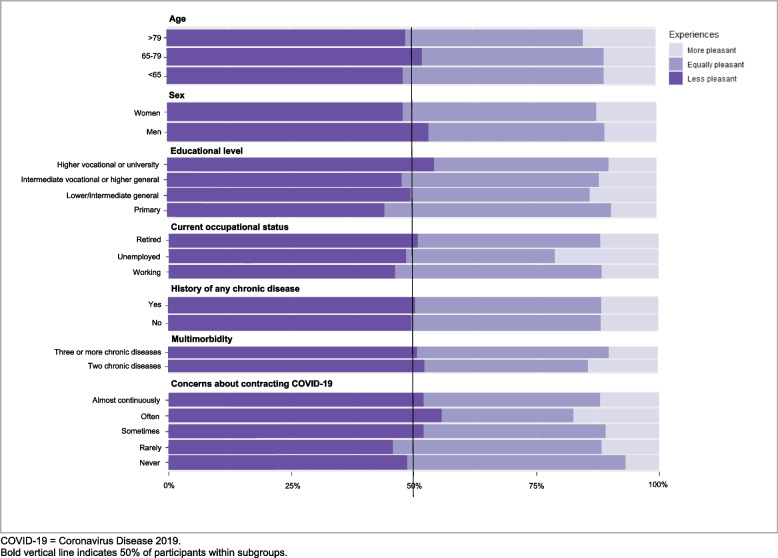
Experiences with virtual consultations compared to in-person consultations. COVID-19 = Coronavirus Disease 2019. Bold vertical line indicates 50% of participants within subgroups

The original article has been corrected and the publisher apologises to the authors and readers for the inconvenience caused by the error.

