# Supplementary material for: Patient perspectives on telemedicine during the COVID-19 pandemic: a mixed-methods community-based study
Source: BMC Health Serv Res. 2023 Jul 27;23:803. doi: 10.1186/s12913-023-09794-w (PMC10375760; doi:10.1186/s12913-023-09794-w)
Supplement: Supplementary file 1 — Supplementary Material 1 [file 12913_2023_9794_MOESM1_ESM.docx]

**Supplementary material**

| Supplementary table 1: characteristics of responders versus non-responders | 2 |
| --- | --- |
| Supplementary table 2: characteristics of excluded participants | 3 |
| Supplementary table 3: perspectives on telemedicine stratified by cardiovascular risk factors/diseases and cancer | 4 |
| Supplementary table 4: characteristics of participants who responded to the question about experiences with telemedicine and of those who also left a free-text comment. | 5 |
| Supplementary table 5: thematic analysis, all (sub)themes and corresponding quotes | 6 |

| **Supplementary table 1. Characteristics of non-responders (N=1350) versus responders (N=4514) to the questionnaire. Values are numbers (percentages) unless stated otherwise.** | | |
| --- | --- | --- |
| **Demographic characteristics** | **Non-responders** | **Responders** |
| **Sex** |  |  |
| Men | 530 (39.3) | 1924 (42.6) |
| Women | 820 (60.7) | 2590 (57.4) |
| **Age (years)** |  |  |
| Mean (SD) | 73.8 (9.2) | 70.3 (11.1) |
| **Educational level** |  |  |
| Primary education | 102 (7.6) | 254 (5.6) |
| Low/intermediate general or lower vocational | 457 (33.9) | 1472 (32.6) |
| Intermediate vocational or higher general | 445 (33.0) | 1433 (31.7) |
| Higher vocational or university | 331 (24.5) | 1304 (28.9) |
| Missing | 15 (1.1) | 51 (1.1) |
| **Both parents born in the Netherlands, yes** | 967 (71.6) | 3612 (80.0) |
| N = number of participants. | | |

| **Supplementary table 2. Characteristics of participants with (N=1103) and without (N=3411) experiences with telemedicine). Values are numbers (percentages) unless stated otherwise.** | | | |
| --- | --- | --- | --- |
| **Demographic characteristics** | **Participants with experiences with telemedicine (N=1103)** | **Participants without experiences with telemedicine (N=3411)** | |
| **Sex** |  | | |
| Men | 456 (41.3) | | 1468 (43.0) |
| Women | (647 (58.7) | | 1943 (56.9) |
| **Age (years)** |  | | |
| Mean, SD | 70.8 (10.5) | | 70.1 (11.2) |
| **Educational level** |  | | |
| Primary education | 54 (4.9) | | 200 (5.9) |
| Low/intermediate general or lower vocational | 352 (31.9) | | 1120 (32.8) |
| Intermediate vocational or higher general | 357 (32.4) | | 1076 (31.5) |
| Higher vocational or university | 328 (29.7) | | 976 (28.6) |
| Missing | 12 (1.1) | | 39 (1.1) |
| **Chronic disease** |  | | |
| No | 153 (13.9) | | 1315 (38.5) |
| Yes | 909 (82.4) | | 1934 (56.7) |
| Missing | 41 (3.7) | | 162 (4.7) |
| **Current occupational status** |  | | |
| Working | 197 (17.9) | | 919 (26.9) |
| On sick leave | 22 (2.0) | | 21 (0.6) |
| Unemployed | 33 (3.0) | | 77 (2.3) |
| Retired | 717 (65.0) | | 2063 (60.5) |
| Other | 77 (7.0) | | 127 (3.7) |
| Missing | 57 (5.2) | | 204 (6.0) |
| **Concerns about contracting COVID-19** |  | | |
| Never | 74 (6.7) | | 552 (16.2) |
| Rarely | 299 (27.1) | | 1075 (31.5) |
| Sometimes | 582 (52.8) | | 1411 (41.4) |
| Often | 115 (10.4) | | 206 (6.0) |
| Almost continuously | 25 (2.3) | | 33 (1.0) |
| Missing | 8 (0.7) | | 134 (3.9) |
| N = number of participants; SD = standard deviation; COVID-19 = Coronavirus Disease 2019. | | | |

| **Supplementary table 3. Experiences with telemedicine compared to physical consultations among participants with cardiovascular diseases and cancer, stratified by type of experience (N=1103). Values are numbers (percentages) unless stated otherwise.** | | | | | |
| --- | --- | --- | --- | --- | --- |
| **Demographic characteristics** | **Full sample (N=1103)** | **More pleasant (N=127)** | **Equally pleasant (N=420)** | **Less pleasant (N=556)** | **p-value** |
| **Cardiovascular disease (any)** |  |  |  |  | 0.597 |
| No | 347 (38.2) | 38 (36.9) | 134 (39.8) | 175 (37.4) |  |
| Yes | 319 (35.1) | 40 (38.8) | 112 (33.2) | 167 (35.7) |  |
| Missing | 242 (26.7) | 25 (24.3) | 91 (27.0) | 126 (26.9) |  |
| **Ever had heart attack** |  |  |  |  | 0.078 |
| No | 597 (54.1) | 68 (53.5) | 236 (56.2) | 293 (52.7) |  |
| Yes | 114 (10.3) | 18 (14.2) | 33 (7.9) | 63 (11.3) |  |
| Missing | 392 (35.5) | 41 (32.3) | 151 (36.0) | 200 (36.0) |  |
| **Narrowing of arteries in the legs** |  |  |  |  | 0.882 |
| No | 615 (55.8) | 73 (57.5) | 231 (55.0) | 311 (55.9) |  |
| Yes | 113 (10.2) | 13 (10.2) | 40 (9.5) | 60 (10.8) |  |
| Missing | 375 (34.0) | 41 (32.3) | 149 (35.5) | 185 (33.3) |  |
| **Ever had stroke/TIA** |  |  |  |  | 0.159 |
| No | 630 (57.1) | 71 (55.9) | 243 (57.9) | 316 (56.8) |  |
| Yes | 111 (10.1) | 18 (14.2) | 34 (8.1) | 59 (10.6) |  |
| Missing | 362 (32.8) | 38 (29.9) | 143 (34.0) | 181 (32.6) |  |
| **Other cardiovascular disease** |  |  |  |  | 0.601 |
| No | 561 (50.9) | 69 (54.3) | 214 (51.0) | 278 (50.0) |  |
| Yes | 150 (13.6) | 18 (14.2) | 51 (12.1) | 81 (14.6) |  |
| Missing | 392 (35.5) | 40 (31.5) | 144 (26.9) | 197 (35.4) |  |
| **Ever had cancer** |  |  |  |  | 0.320 |
| No | 671 (60.8) | 81 (63.8) | 251 (59.8) | 339 (61.0) |  |
| Yes | 255 (23.1) | 24 (18.9) | 107 (25.5) | 124 (22.3) |  |
| Missing | 177 (16.0) | 22 (17.3) | 62 (14.8) | 93 (16.7) |  |
| N = number of participants; COVID-19 = Coronavirus Disease 2019. | | | | | |

| **Supplementary table 4. Characteristics of participants who responded to the question about experiences with telemedicine and of those who also left a free-text comment. Values are numbers (percentages) unless stated otherwise.** | | |
| --- | --- | --- |
| **Demographic characteristics** | **Of full sample (N=1103)** | **Of the subsample who left a free-text comment (N=752)** |
| **Sex** |  |  |
| Men | 456 (41.3) | 301 (40.0) |
| Women | 647 (58.7) | 451 (60.0) |
| **Age (years)** |  |  |
| Mean, SD | 70.8 (10.5) | 69.2 (10.4) |
| **Educational level** |  |  |
| Primary education | 54 (4.9) | 33 (4.4) |
| Low/intermediate general or lower vocational | 352 (31.9) | 219 (29.1) |
| Intermediate vocational or higher general | 357 (32.4) | 244 (32.4) |
| Higher vocational or university | 328 (29.7) | 251 (33.4) |
| Missing | 12 (1.1) | 5 (0.7) |
| **Chronic disease** |  |  |
| No | 153 (13.9) | 98 (13.0) |
| Yes | 909 (82.4) | 635 (84.4) |
| Missing | 41 (3.7) | 19 (2.5) |
| **Occupational status** |  |  |
| Working | 197 (17.9) | 156 (20.7) |
| On sick leave | 22 (2.0) | 18 (2.4) |
| Unemployed | 33 (3.0) | 28 (3.7) |
| Retired | 717 (65.0) | 464 (61.7) |
| Other | 77 (7.0) | 58 (7.7) |
| Missing | 57 (5.2) | 28 (3.7) |
| **Concern contracting COVID-19** |  |  |
| Never | 74 (6.7) | 46 (6.1) |
| Rarely | 299 (27.1) | 207 (27.5) |
| Sometimes | 582 (52.8) | 400 (53.2) |
| Often | 115 (10.4) | 79 (10.5) |
| Almost continuously | 25 (2.3) | 16 (2.1) |
| Missing | 8 (0.7) | 4 (0.5) |
| N = number of participants; SD = standard deviation; COVID-19 = Coronavirus Disease 2019. | | |

| **Supplementary table 5 – Thematic analysis, all (sub)themes and corresponding quotes.** | | | |
| --- | --- | --- | --- |
| **Theme** | **Number of comments (N=752)** | **Subtheme** | **Supporting quote** |
| **Less pleasant** | **N (%)** |  |  |
| Lack of nonverbal communication | 233 (30.9) | Unable to interpret nonverbal communication | *“You cannot see someone’s facial expressions and body language. The other person cannot see it from me either. I think that is a huge source of information.*” (man, 62 years) |
|  |  | Lack of personal contact | *“A face-to-face conversation is part of my care. I experience a telephone conversation as cold.”* (man, 81 years)  *“It is a lot more impersonal. I want to be able to look someone in the eyes.”* (woman, 69 years) |
| Lack of physical examination | 127 (16.9) | Usual tests could not be performed | *“My eye pressure, sight and any further damage to the optic nerve could not be measured through the phone.”* (woman, 65 years)  *“I received blood pressure lowering medication, without having my blood pressure checked.”* (man, 80 years)  *“I have breast cancer, and my doctor was unable to feel and look at my breasts.”* (woman, 77 years) |
|  |  | Less trust in proposed treatment plan/diagnosis | *“Some physical symptoms are hard to explain if you cannot point to them. The medical specialist cannot feel or assess the complaint.”* (woman, 50 years)  *“Now I had to tell the doctor if I noticed any changes myself. Very subjective.”* (woman, 67 years) |
| Somatic and/or language barriers | 68 (9.0) | Speech, hearing, or concentration issues; language barrier | *“I am partially deaf (vestibular schwannoma) and cannot quite understand the conversation over the phone. And then I’ll just let it run its course.”* (man, 88 years)  *“I do not speak Dutch very well.”* (woman, 62 years) |
|  |  | Less assertive | *“I cannot take my time and think of questions to ask, or respond to information that the medical specialist usually shows me on a computer screen. I ask fewer questions while I want to know more.”* (woman, 65 years) |
| Consultation scheduling | 53 (7.0) | Unsatisfied duration conversation | *“I had the impression that the medical specialist rushed the conversation. Afterwards I thought of questions that I still wanted to ask.”* (man, 66 years) |
|  |  | Time consultation unclear, or not able to discuss specific time | *“It is not clear when the doctor will call – early or late in the afternoon. I am at home waiting all day for the phone call.”* (woman, 81 years)  *“The phone call caught me by surprise, I was not well prepared for the consultation.”* (man, 67 years) |
| Acquaintance with physician | 15 (1.9) | Unpleasant if unfamiliar with physician | *“I have a new ophthalmologist and would have appreciated meeting him in person.”* (woman, 82 years) |
|  |  | Physician struggling with technology | *“Not all of them were able to deal with video calls.”* (woman, 61 years) |

| **Equally pleasant** |  |  |  |
| --- | --- | --- | --- |
| Comparable quality | 55 (7.3) | No clear differences in consultation content | *“The general practitioner paid just as much attention to me as during an in-person consultation.”* (woman, 56 years)  *“I was asked the exact same questions as face-to-face.”* (woman, 57 years) |
| Symptom-dependent | 40 (5.3) | Severity of symptoms | *“I can imagine that if we would need to discuss something serious, an in-person conversation would be more pleasant.”* (man, 64 years) |
| Alternation preference | 19 (2.5) | Varying between in-person and telephone consultations | *“I was well listened to, and subsequently it was decided that I had to come to the hospital to get blood drawn to see if there were any additional problems.”* (woman, 54 years) |
| **More pleasant** |  |  |  |
| Consultation content | 140 (18.6) | No need for physical consultation | *“It was just an annual check-up that could easily be handled through the phone.”* (man, 84 years) |
|  |  | Convenient for discussing results, or asking short questions | *“I did not have to leave my house: discussing results of blood tests can also be done by phone.”* (man, 74 years) |
| Personal circumstances | 107 (14.2) | No need to travel, leave home or work | *“I was sitting in my own safe environment with my partner beside me.”* (woman, 72 years)  *“It saves a lot of travel time, and parking costs in particular, these keep getting higher and higher.”* (woman, 68 years) |
|  |  | Mobility issues | *“I don’t have to walk as much, I have COPD.”* (man, 81 years) |
| Satisfaction with physician | 36 (4.8) | Physician was prepared and showed involvement | *“The medical specialist took enough time, read my file properly, and came up with a proposal that I completely agreed with*.” *(man, 66 years)* |
| COVID-19 | 26 (3.5) | No risk of infection | *“The phone call is for my own protection. The healthcare provider is there for me despite the corona crisis.*” (woman, 59 years) |
